# Recent Advances in Synthesis and Degradation of Lignin and Lignin Nanoparticles and Their Emerging Applications in Nanotechnology

**DOI:** 10.3390/ma15030953

**Published:** 2022-01-26

**Authors:** Virendra Kumar Yadav, Nitin Gupta, Pankaj Kumar, Marjan Ganjali Dashti, Vineet Tirth, Samreen Heena Khan, Krishna Kumar Yadav, Saiful Islam, Nisha Choudhary, Ali Algahtani, Sweta Parimita Bera, Do-Hyeon Kim, Byong-Hun Jeon

**Affiliations:** 1 Department of Microbiology, School of Sciences, P P Savani University, Surat 394125, India; virendra.yadav@ppsu.ac.in (V.K.Y.); sweta.bera@ppsu.ac.in (S.P.B.); 2 School of Nano Sciences, Central University of Gujarat, Gandhinagar 382030, India; nitinkgupta1988@gmail.com; 3 Integrated Regional Office, Ministry of Environment, Forest and Climate Change (MoEFCC), Government of India, Saifabad, Hyderabad 500004, India; pankajb434@yahoo.com; 4 Department of Biological Sciences, University of Texas at Dallas, 800 W Campbell Road, Richardson, TX 75080, USA; ganjali_marjan@yahoo.com; 5 Mechanical Engineering Department, College of Engineering, King Khalid University, Abha 61421, Saudi Arabia; vtirth@kku.edu.sa (V.T.); alialgahtani@kku.edu.sa (A.A.); 6 Research Center for Advanced Materials Science (RCAMS), King Khalid University, Guraiger, P.O. Box 9004, Abha 61413, Saudi Arabia; 7 Research and Development Centre, YNC Envis Pvt. Ltd., New Delhi 110059, India; samreen.heena.khan@gmail.com; 8 Faculty of Science and Technology, Madhyanchal Professional University, Ratibad, Bhopal 462044, India; envirokrishna@gmail.com; 9 Civil Engineering Department, College of Engineering, King Khalid University, Abha-61421, Saudi Arabia; sfakrul@kku.edu.sa; 10 Department of Environment Science, School of Sciences, P P Savani University, Surat 394125, India; nishanaseer03@gmail.com; 11 Department of Earth Resources and Environmental Engineering, Hanyang University, Seoul 04763, Korea; kimdohyeon@hanyang.ac.kr

**Keywords:** lignin, nanoparticles, ligninolytic enzymes, degradation, nanobioremediation

## Abstract

Lignin is an important commercially produced polymeric material. It is used extensively in both industrial and agricultural activities. Recently, it has drawn much attention from the scientific community. It is abundantly present in nature and has significant application in the production of biodegradable materials. Its wide usage includes drug delivery, polymers and several forms of emerging lignin nanoparticles. The synthesis of lignin nanoparticles is carried out in a controlled manner. The traditional manufacturing techniques are costly and often toxic and hazardous to the environment. This review article highlights simple, safe, climate-friendly and ecological approaches to the synthesis of lignin nanoparticles. The changeable, complex structure and recalcitrant nature of lignin makes it challenging to degrade. Researchers have discovered a small number of microorganisms that have developed enzymatic and non-enzymatic metabolic pathways to use lignin as a carbon source. These microbes show promising potential for the biodegradation of lignin. The degradation pathways of these microbes are also described, which makes the study of biological synthesis much easier. However, surface modification of lignin nanoparticles is something that is yet to be explored. This review elucidates the recent advances in the biodegradation of lignin in the ecological system. It includes the current approaches, methods for modification, new applications and research for the synthesis of lignin and lignin nanoparticles. Additionally, the intricacy of lignin’s structure, along with its chemical nature, is well-described. This article will help increase the understanding of the utilization of lignin as an economical and alternative-resource material. It will also aid in the minimization of solid waste arising from lignin.

## 1. Introduction

Lignin is one of the main renewable sources of aromatic molecules and is regarded as the second-largest renewable source. After cellulose, it is the best prolific polymer to procure carbon [1]. It gives rise to about 30% of woody plant tissues and is involved with the cross-connecting of cellulose. It gives strength, inelasticity and firmness to cell divisions. Lignin is a highly complex compound for enzymatic depolarization as it is insoluble in water, is artificially convoluted and lacks hydrolyzable linkages [2]. Lignin constituents are an important component of plant litter input, which constitutes nearly 20% of the soil [3,4]. It is an amorphous, 3-dimensional polymer, whose shape represents its material-binding property [5]. In plant cell walls, lignin is a part of the lignocellulosic compound, which consists of 40–60% cellulose, 20–40% hemicelluloses and 10–25% lignin [5,6]. Globally about 50 million tons of lignin are manufactured annually by the paper and pulp industries, with around 2% recuperated for its application in chemical manufacturing [7,8]. The interest in lignin has drastically increased in the scientific and industrial community over the last decade. The reason for this is the growing concern over climate change and the fundamental need to decrease industrial pollution [9]. 

Due to the complex structure of lignin, it cannot be degraded by most degradation methods, which makes it a recalcitrant material and poses a challenge that needs to be resolved [10]. The degradation of lignin improves the earth’s biofuel resources and would also provide a substitute for the harsh technologies that are implemented in industries such as the paper and pulp sector [11]. The key lignin-degradation investigations are based on biotic, oxygen-dependent and co-metabolic methods. Many researchers have already proven that specific microorganisms, such as bacteria and certain fungi, are capable of biodegrading different biopolymers in soil [12]. For example, wood-rotting basidiomycetes fungi can degrade lignin. During the degradation of lignocellulosic biomass (LCBM), three common lignin peroxide enzymes are secreted: laccase (Lac), manganese peroxidase (MnP) and lignin peroxidase (LiP) [13]. Additionally, lignin degradation was also observed in *Phanerochaete chrysosporium* crust fungi culture [14]. The degradation of lignin by microbes such as white-rot fungi (WRF) occurs under conditions of biodegradation that acquire a great oxidative order with some substrate specificity [15]. Certain fungi, as well as their extracted enzymes, have shown increased biodegradation of lignin, and may be especially applicable to industries such as pulp, agriculture, paper and bioremediation [16].

LCBM has been accredited for prospective use to produce chemicals and various biomaterials [17]. The inclusion of lignin is a great challenge in the current scenario. Several investigations have been performed to increase lignin utilization in value-added applications [18]. Lignin is considered an ideal component for the preparation of a various range of chemicals due to its abundant sources and inexpensiveness. Lignin is a by-product produced by paper mills and lignocellulose feedstock biorefineries. In paper mills, 98% of scientific lignin is consumed internally for energy restoration and about 2% is applied economically [19]. It is essential to degrade lignin completely in order to prepare different chemicals. However, there are several challenges in preparing chemicals from lignin. The degradation of lignin can be performed by chemical, thermal or enzymatic pathways [20,21,22].

Lignin is comprised of various functional groups, such as hydroxyl (-OH), methoxy (CH_3_-O), carbonyl (-C=O-), carboxyl (-COOH) and benzene (C_6_H_6_) [23], which makes it a suitable material for numerous applications. Many researchers are trying to combat the challenges associated with transforming lignin into different valuable products by developing diverse procedures and pathways. They are exploring different research studies and production techniques together in order to make lignin a precious raw material. Lignin can also have a very crucial role in petroleum refineries, as it can supply a continual source for the preparation of valuable aromatic molecules [24]. Europe, the USA and Canada are manufacturing various types of lignin products, such as adhesives, binders and chemicals. Some Asian countries, such as India, Japan and China, are also creating lignin-derived products. China is presently the leader in lignin-based chemical production among all the countries [25]. 

Bulk and composite materials of lignin have limited application. Moreover, if they are produced from expensive precursors then the final material itself will be very expensive. Therefore, the use of lignin waste to synthesize lignin nanoparticles will drastically revolutionize the lignin production cycle. There are several reports where lignin or lignin composites have been developed and used for various applications. Recently, Bonilla and Bonilla, 2021 reported the synthesis of a lignin-based biopolymer from gorse (*Ulex europaeus*), which is an invasive plant [26]. Larrenata et al., 2018 reported the synthesis of lignin hydrogels by combining LiG with poly(ethylene glycol) and poly(methyl vinyl ether-co-maleic acid) by an esterification process, where the synthesis was carried out in a solid state and could be accelerated by using microwave (MW) radiation. The hydrophobic nature of the LiG helped in the loading of the hydrophobic drug curcumin. Moreover investigators also found that the LiG had antimicrobial properties due to its macromolecule nature, with antimicrobial activity observed against *Staphylococcus aureus* and *Proteus mirabilis* [27]. Liangliang An et al., reported the synthesis of tailor-made zwitterionic lignin with protein resistance by applying a two-step grafting reaction. The LiG was modified by using 3-Dimethylamino-1-propyl chloride hydrochloride and 1,3-Propanesultone and confirmation was performed by multiple instruments. The synthesized LiG had negative zeta potential, indicating hydrophilicity [28]. Spiridon et al., 2021 reported the synthesis of new ferrite lignin-hybrids, which were obtained by the combustion of cobalt nitrate and ferric nitrate, two types of LiG being used as combustion agents at calcination temperature of 500 °C and 900 °C, respectively. Further, the authors investigated the synthesized material by using Fourier-transform infrared spectroscopy (FT-IR), X-ray powder diffraction (XRD), scanning electron microscopy (SEM), energy-dispersive X-ray (EDX), X-ray photoelectron spectroscopy (XPS) and vibrating sample magnetometer system (VSM) for magnetic properties. This particular hybrid had a spinel structure whose crystal size increased with greater calcination temperature [29]. Kumar et al., 2021 has used LiG particles for gene/drug delivery and for tissue-engineering applications [30].

Nanoparticles and nanotechnology have played an immense role in the field of drug delivery, medicine, research and environmental cleanup [31,32,33,34,35]. Due to their high surface-area-to-volume ratio and high surface energies they are considered much more useful than bulk materials [34,36,37,38,39]. Thus various LiG NPs, either synthesized chemically in the laboratory or derived from lignin-rich waste, are increasingly used in day-to-day life. For instance, due to their structural diversity and biodegradability, LiG NPs have emerged as a promising alternative for some of the value-added materials traditionally created from fossil fuel-based chemicals and products. 

Understanding the detailed environmental biodegradation mechanism will help in lignin’s utilization and will also minimize solid waste accumulation [40]. Very little information is available in the scientific domain about the application of surface-functionalized lignin and lignin nanoparticles. The expense of lignin precursors and synthesis routes have restricted lignin and lignin nanoparticles to scientific applications. Thus there is need to develop new methods for the synthesis of lignin particles which are biodegradable/compostable and/or recyclable, from lignin-rich waste, which would otherwise accumulate in the environment. Despite significant efforts in producing value-added products, lignin is still underutilized, and future, integrated biorefineries strongly depend on the ability to effectively isolate, purify and utilize lignin [41]. Debate continues on which microbes use lignin as a source of carbon, as detailed information from scientific studies does not exist. One such recent study carried out by Cerro et al., 2021, where the role of WRF in the utilization of lignin as a carbon source has been controversial [42]. White rot fungi have been shown to be more efficient lignin degraders than soft rot fungi, but detailed explanation supported by scientific evidence is lacking. Complete biodegradation of brown-rotted wood has not yet been achieved. The current review work seeks to address these gaps in research.

The current review work emphasizes the state-of-the-art, latest advancements in the synthesis of lignin micro- and nanoparticles by physical, chemical and biological routes. Moreover, this review work also emphasizes the various microbial approaches for the remediation of lignin in the environment and in simulated laboratory conditions. Another objective of this review work is to provide the latest biodegradation pathways for mineralization by various fungi and bacteria. Finally, this review provides emerging applications for lignin and lignin nanoparticles in research, environmental cleanup and biodegradable materials. The suggested applications will help reduce pollution arising from the accumulation of non-degradable material in our environment.

## 2. Structure and Biosynthesis of Lignin

**Figure 1:**
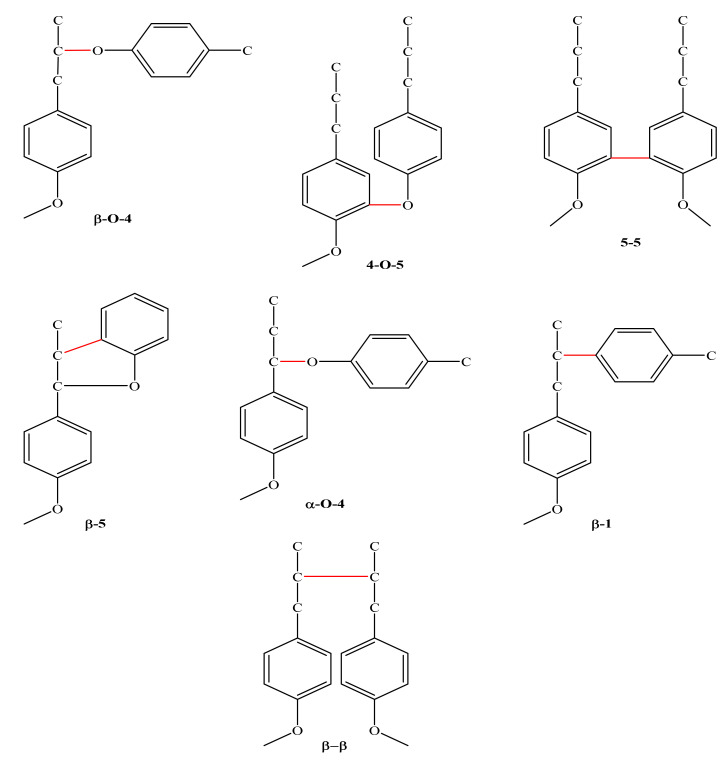
Molecular structure representation of bonds present in lignin molecules.

Lignin is a well-branched phenol polymer and accounts for about 15–20% of the total weight of LCBM [43]. Based on its molecular structure, lignin is made up of three main components that are connected by a variety of bonds [29]. As per the nature of LCBM, the structure of lignin is also variable, and the process of degradation powerfully dependent on its constitution. Proper structural characterization is essential for organization and usage of lignin. Efficient lignin degradation has traditionally been the major technique for the analysis of its molecular structure. Various advanced spectroscopic analysis techniques, such as ultraviolet spectroscopy (UV-Visible), Fourier-transform infrared spectroscopy (FTIR), Raman spectroscopy and nuclear magnetic resonance (NMR) spectroscopy, can give both qualitative and quantitative data on functional groups, bonds, characterization of molecular structure and constitutional properties of lignin and its degradation products [44,45]. Different NMR methods, such as proton NMR (1H), carbon-13 (13C), 19F and 31P plus 2D-NMR, are used for the complete study of the structure of lignin [45]. Quantitative 13C-NMR and a range of 2D-NMR approaches provide both qualitative and quantitative data for the complete lignin [46]. In the plant cell, lignin biosynthesis takes place via the arrangement of three principles: hydroxycinnamoyl, alcohol monomers or monolignols (MLGs: p-coumaryl alcohol, coniferyl alcohol (CA) and sinapyl alcohol (SA)). These MLGs are typically denoted as phenylpropanoids (PP), which vary in the compositions at the 3- and 5-C positions in the aromatic ring structure. The preparation of lignin begins with the random polymerization of a phenoxy radical coupling that produces an oligomeric yield [47]. After the polymerization process, these polymers are known as p-hydroxyphenyl (H), guaiacyl (G) and syringyl (S) for p-coumaryl alcohol [48]. The MLGs are attached by ether bonds. The various molecular bonds present in lignin molecules are given in Figure 1.

## 3. Microbial Synthesis of Lignin Nanoparticles (LNPs)

The production of nanomaterials using physicochemical techniques can cause toxic conditions in the surrounding environment and sometimes requires costly materials and equipment. The application of economically feasible, simple, safe and environmentally friendly methods for the synthesis of nanomaterials that comply with green chemistry is vital [49]. The synthesis of lignin nanoparticles (LiG NPs) is a good approach for enhancing the blending behavior of lignin. These synthesized LiG NPs have new and enhanced behavior compared to the original materials [50]. LiG NPs can be suitable nanosized components for several biomaterial applications due to their smooth structures and stability in various physiological conditions. The nanosized form of lignin can also overcome the hurdles faced by bulk lignin particles due to their heterogeneity and decreased water solubility [51]. 

## 4. Different Methods for LiG NPs Synthesis 

**Figure 2:**
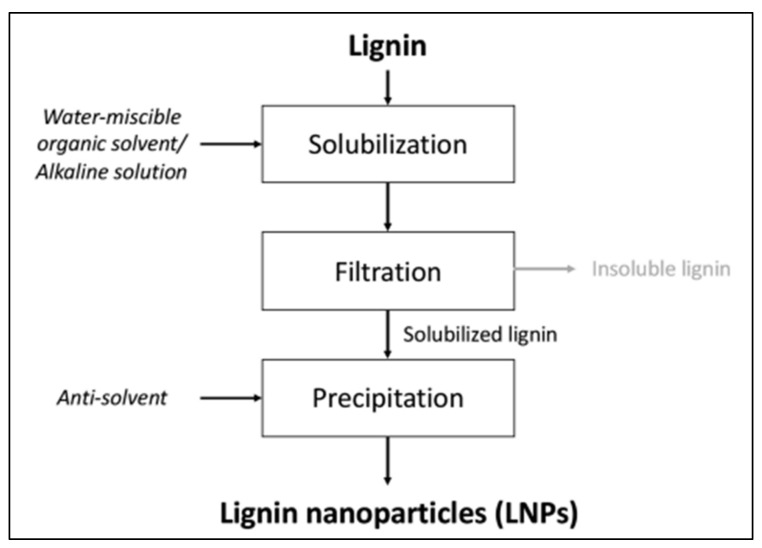
Schematic diagram for synthesis of LiG NPs by chemical routes, adapted with permission from Meng et al., 2021 [41], American Chemical Society.

Different chemical methods, such as CO_2_ saturation, solvent exchange, ultrasonication, continuous solvent exchange, sonication, dialysis, water-in-oil micro-emulsion, acid precipitation, self-assembly, interfacial cross-linking and emulsion, freeze-drying, thermal stabilization, homogenization and microbial and enzyme-mediated, have been developed to synthesize LiG NPs from bulk lignin particles [52,53,54,55,56,57,58,59,60,61,62,63]. All of these chemical methods have a common approach, which is shown schematically in the Figure 2. Here we will discuss some of the frequently applied methods used to produce LiG NPs. 

### 4.1. Acid Precipitation

This unique method is used to synthesize LiG NPs that are non-toxic for yeast and microalgae [64]. Researchers have described two different methods, which result in different stability of particles within mediums having varying pH ranges (low pH and high pH). In the first method, there is precipitation of low-sulfonated lignin by using a solution of ethylene glycol prepared in a dilute acidic-aqueous solution, which produces LiG NPs that maintain higher stability at various ranges of pH. In the second method of acidic precipitation the LiG NPs were synthesized in a high-pH aqueous solution. These LiG NPs showed stability only at an acidic pH [65]. 

### 4.2. Ultrasonication

The ultrasonication method falls in the category of mechanical techniques. Similar to other mechanical techniques, in this technique the size of the bulk particles is reduced down to as small as the nanometer (10^−^^9^ m) range. Yet inconsistency in size and a broad particle-size distribution are the principal impediments of this strategy. However, due to its simplicity this process is preferred by the NP manufacturing industries and research communities. This technique is also used for the synthesis of LiG NPs and other types of nanomaterials, such as carbon-based nanoparticles (graphene oxide nanosheets, etc.) In 2015, Gilca et al. used ultrasonic irradiation to obtain LiG NPs from wheat straw and sarkanda grass [40]. Additionally, by using several characterization techniques, it has also been confirmed that ultrasonication at the intensity applied for breaking down bulk particles is not responsible for changes in either the structure or composition of the synthesized LiG NPs [66,67,68].

### 4.3. Self-Assembly

In this method, an uncertain arrangement of pre-existing components produces either an agglomerated, spherical-shaped pattern or an organized structure and a stable nano-sized particle. A spherical-shaped pattern is created due to specific non-covalent interactions with the lack of any external direction. In this method, the nature of the solvent and various non-covalent interaction forces play a significant role in the agglomerate and self-assembled of LiG NPs. The significant non-covalent interaction that forces their bond energies are π-π interactions (4 to 30 kJmol^−1^), hydrogen-bonding (4 to 30 kJmol^−1^), van der Waals forces (1 to 4 kJmol^−1^) and chain entanglement [65,69]. In the self-assembly process, cyclohexane (CHX) is incorporated into the lignin solution of alkaline pH. It is then re-dissolved in dioxane. With the incorporation of CHX, flocculation as well as precipitation of lignin micelles occurs. In 2019, Mishra and Ekielski published research related to the synthesis of self-assembly of lignin (molecular and supramolecular) NPs by using a simple spray freezing method [59]. In this work, dimethyl sulfoxide solvent was used because of its two unique properties: its solubility of lignin and the high boiling point of the solvent.

### 4.4. Solvent Exchange Method

Solvent exchange is a straightforward system with a vast range of applications. In this technique, the water-miscible organic solvent is used to prepare a solution of an organic compound, and then excess water is mixed with it. After that, nanoparticles are produced due to their decreased solubility. In one research study, dioxane LiG NPs and alkali LiG NPs were synthesized by the utilization of the nanoprecipitation method from two different sources of lignin: hardwood dioxane lignin and softwood alkali lignin. The fabricated dioxane LiG NPs and alkali LiG NPs were analyzed by various advanced microscopy techniques. The results revealed that both types of NPs had a spherical shape with a size between 80–104 nm [70].

### 4.5. Biological Methods

**Figure 3:**
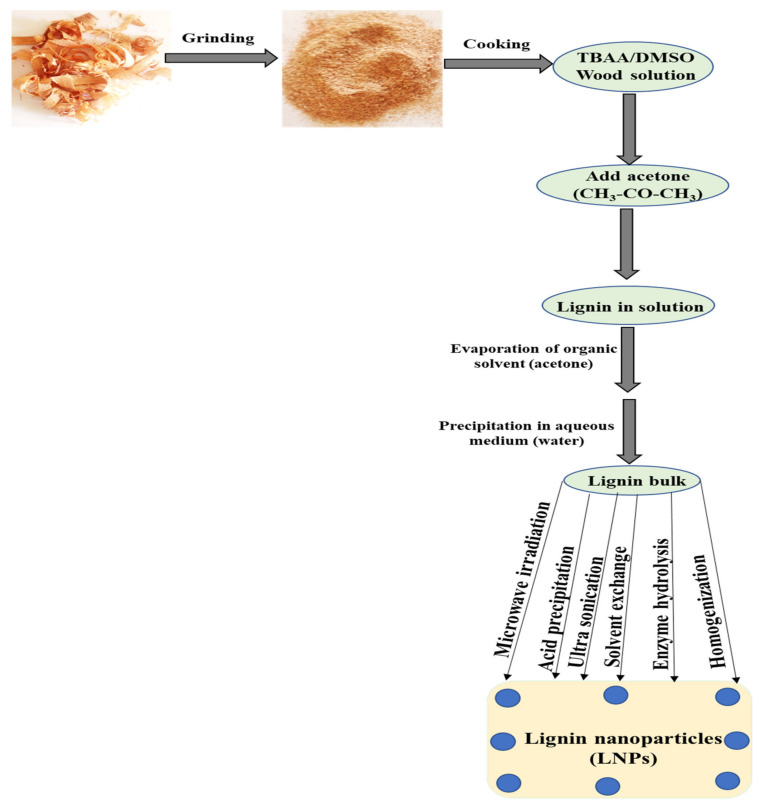
Schematic diagram of various methods for LiG NP synthesis.

The transformation of lignin into LiG NPs by enzymatic hydrolysis improves the physicochemical properties of lignin bulk. In 2017, Rangan and colleagues were the first to synthesize lignin-rich cuboidal nanoparticles using lignocellulosic fibers isolated from Indian ridge gourd by using specific enzymes to crush the LC complex [71]. Microscopic analysis of data revealed that the size of the nanoparticles varied between 20–100 nm with consistent morphology. In another study, researchers isolated *Aspergillus oryzae*, a mold-filamentous fungus, to obtain nanolignins. About 45.3% nanolignin was produced from the microbial strain, 79.50% from homogenization and 62.60% from ultrasonication. The microbially synthesized nanolignin exhibited antibacterial, antioxidant and ultraviolet-resistant characteristics on the outer surfaces of cotton and linen fabrics [72]. Synthesis of LiG NPs by an enzymatic cross-linking method using laccases could be a very effective technique. Fungal laccase enzymes were purified from two different fungi (*Trametes hirsute (ThL)* and *Melanocarpus albomyces (MaL)*) and were further utilized in the copolymerization processes for LNP synthesis. The LiG NPs could be dried and resuspended in tetrahydrofuran (THF) or water to retain the stable property of LiG NPs, such as their size and shape. The reactivity of ThL and MaL on Lignoboost lignin and LiG NPs were analyzed and confirmed by high-performance, size-exclusion chromatography and oxygen-utilization measurements by synchronous recognition of red-brown color due to the development of quinine [57]. Figure 3 shows the synthesis of LiG NPs from various sources.

### 4.6. Flash Nano Precipitation (FNP)

Copolymer stabilization is used to prepare nanoparticles in this method. The FNP method is used for nanoparticle formulation for applications such as drugs and imaging agents as it provides high solute loading and high encapsulation efficiencies. Most importantly, green solvents can be used for the synthesis of lignin nanoparticles, which makes this method ecofriendly and sustainable [73]. Conner et al., 2020, reported the synthesis of concentrated monodisperse LiG NPs by recirculation-enhanced FNP method. It was found that LiG NPs were formed by continuous burst nucleation at the time of mixing, and that there was no diffusive growth. A consequence of this technique was a highly uniform and controlled size, followed by a modified LaMer nucleation and growth mechanism. Further, they concluded that highly stable and uniformly sized LiG NPs could be synthesized in large volume by the FNP method, and that it could be used for biological as well as agricultural applications [74].

## 5. Biodegradation of Lignin

Several extracellular enzymes, such as lignin peroxidase, manganese peroxidase, laccase, cellobiose and quinine oxidoreductase, have been extracted and purified from ligninolytic fungi. Among these, the first three enzymes have oxidative and reductive natures. Various studies are currently seeking to elucidate the key function of these enzymes, and how they degrade lignin through a biological process [2]. The degradation of LCBM by a biological method is environmentally friendly and inexpensive. The lignin in tobacco stalk is degraded by fungi belonging to *Moniliales gliocephalias* sp. and *Aspergillus* sp. [14]. However, the microbes which are involved in this biodegradation process are limited due to the higher concentrations of lignifications and nicotine in the tobacco stalk [14]. Techniques based on biological methods for the depolymerization and metabolism of the lignin show the potential to reduce the cost of lignocellulosic biorefining. The pretreatment method enhances downstream processing and cellulosic fermentation, making an important outflow stream of aromatic compounds for the production of valuable products [75]. Currently, lignin effluent is also used for combustion and internal energy creation in biorefineries, with a 60% surplus supply [2]. Among different fungi, basidiomycetes are the only fungi that facilitate wide biodegradation of lignin. (Though certain white-rot and brown-rot fungi have the ability to completely mineralize and modify lignin, respectively, when the carbohydrates have been removed from the wood [42].)

## 6. Lignin Degradation in Soil

Lignin is considered an important ingredient of soil organic matter. Additionally, it helps to reserve the carbon dioxide in soil [2]. However, its complex structure and unmanageable nature make lignin’s degradation an essential task. From ancient times, various scientists and researchers have tried to understand the long-chain polymeric complex structure, and several attempts have been made to develop the easiest, eco-friendliest and cheapest methods to degrade it. Several methodologies are currently applied in the paper and pulp industries to degrade lignin and/or to use it for the manufacture of biofuels. However, these methods present several difficulties, and produce lower yields of biofuel than other strategies. For overcoming these problems, plus increasing biofuel production, certain bacteria and fungi can be used to break down a variety of lignin biopolymers in soil [76]. Enzymes that are isolated from specific species of bacteria and fungi are applied to catalyze several reactions, such as oxidation and hydroxylation, depolymerization of phenolic as well as non-phenolic polymers of lignin, and the mineralization of insoluble lignin [77]. The degradation rate of lignin in the soil is directly affected by the induction, adsorption and diffusion of the ligninolytic enzymes [12]. In the last decade, various studies have been performed on the degradation of LCBM by using biological methods, especially in wood-rotting basidiomycetes microorganisms (i.e., white-rot and brown-rot fungi). Among the wood-rot basidiomycetes microorganisms, the most effective biodegraders of LCBM are white-rot fungi (WRF) (e.g., *Penicillium chrysosporium*). These fungi have also shown a higher degradation rate for lignin (faster than brown-rot fungi and other microorganisms) [78]. In 2016, the rate of degradation of lignin in tobacco stalk by *P. chrysosporium* was investigated; it was found that around 53.57% of the lignin was degraded in 15 days [14]. Numerous extracellular enzymes are released by white-rot fungi. These enzymes solely function to decompose the lignin, cellulose and hemicellulose that is present in the plant cell wall [62]. These enzymes include laccases and peroxidases, such as LiP, MNP and versatile peroxidase (VP). Laccases and peroxidases degrade lignin via free radicals of lower molecular weight, such as hydroxyls (OH), that depolymerize the lignin polymers containing phenolic and non-phenolic groups, and also mineralize insoluble lignin [2]. Lignin degradation by brown-rot fungi (BRF) generally includes oxidation reactions by non-enzymatic methods, which produce -OH radicals by Fenton chemistry [79]. BRF leads to partial oxidation of lignin via demethylation of the aromatic ring [80]. During this cycle, the phenolic -OH composition of the reaction mixture increases due to partial oxidation and incorporation of new carboxyl and carbonyl functional groups [81].

## 7. Lignin-Degrading Enzymes

**Figure 4:**
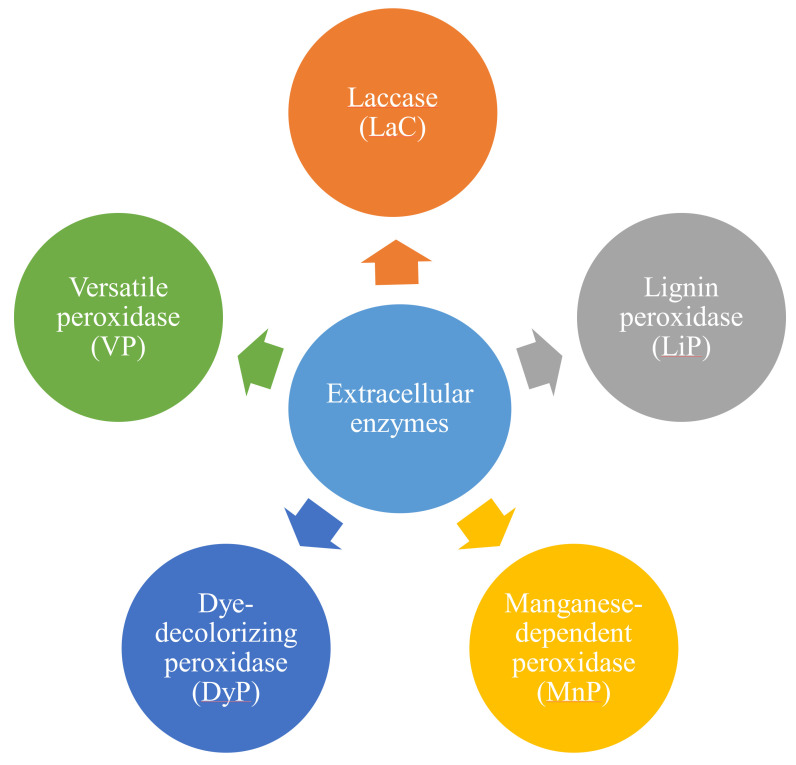
Different types of extracellular enzymes involved in lignin degradation.

Specific species of bacteria and fungi release numerous enzymes that can catalyze many oxidative and hydroxylation reactions [82]. LCBM biodegradation was investigated in wood-rotting basidiomycetes microorganisms [76]. The enzyme-mediated degradation of lignin consists of five different extracellular enzymes, which are shown below in Figure 4.

Lacasse catalyzes the oxidation reaction of polyphenols and methoxy-substituted phenols by generating free radicals [83]. The reduction of molecular oxygen to water leads to oxidation. Laccase enzyme can evenly catalyze and disintegrate the non-phenolic lignin structures, including the dissolution of β-O-4 linkages in the occupancy of redox mediators [84]. Another enzyme, lignin peroxidase, uses hydrogen peroxide (H_2_O_2_) as the oxidizing agent for the oxidative depolymerization of lignin [85]. These enzymes are generally not specific, and, as a result, they oxidize various phenolic aromatic compounds and a variety of non-phenolic lignin compounds. The manganese peroxidase enzyme makes use of H_2_O_2_ to oxidize Mn^2+^, which is readily available in wood and different soils, therefore producing Mn^3+^ ions [86]. The latter is stabilized by chelators and behaves as diffusive charge-transfer intermediates that are capable of oxidizing phenolic substrates. The VP enzyme merges the properties of LiP and MnP enzymes, with their multiple methods of catalytic efficacy reflected in their name [87]. Not only do they oxidize Mn^2+^ to Mn^3+^, similar to MnP enzyme, but they are also capable of oxidizing non-phenolic compounds in the same manner as LiP enzyme. The DyP-type peroxidase enzyme, however, has different series, structures and utility, and does not conform to the characteristics classically associated with plant/microbial peroxidase [88]. The DyP-type peroxidase enzyme potentially degrades lignin. It is capable of oxidizing dyes, non-phenolic lignin compounds, such as veratryl alcohol, and β-O-4 linkages [1].

## 8. Steps Involved in Lignin Degradation

**Figure 5:**
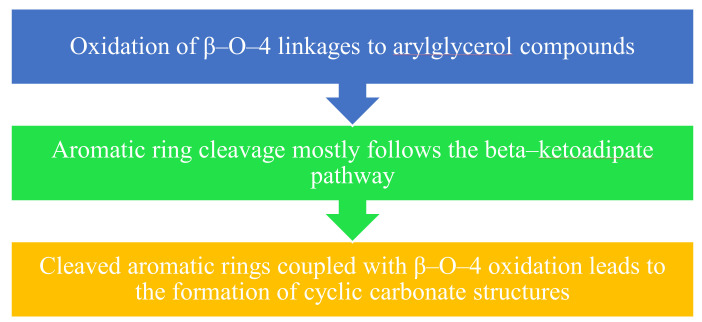
Oxidation of lignin by extracellular enzymes.

Lignin biodegradation involves both depolymerization and aromatic ring cleavage [89]. Extracellular enzymes facilitate oxidation of lignin in the following steps, as shown in Figure 5.

There are several hypotheses which purport the formation of humic acid from lignin. First, the lignin is broken down into several smaller constituents, and then the smaller particles reassemble to create a complex organic compound. Additionally, lignin can also be broken down by enzymatic combustion. Enzymatic combustion is a process where there is the formation of reactive intermediates from the enzyme, but there is no direct control of the reaction [12]. Several economically important processes depend on lignin decomposition, for instance decay of wood and biogeochemical cycling of woody biomass.

## 9. Lignin Degrading Fungi

**Figure 6:**
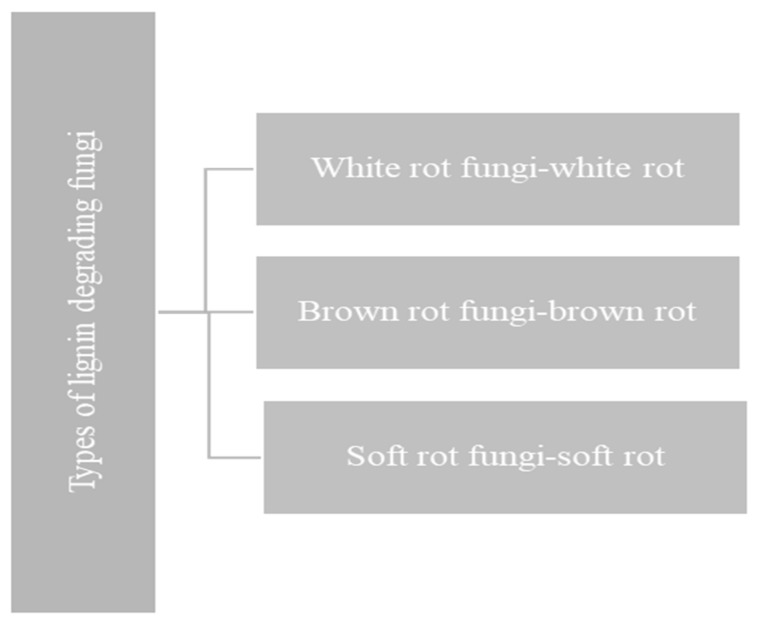
Types of fungi involved in the degradation of lignin.

The fungal breakdown of lignocelluloses depends upon two types of extracellular enzymes. First, the fungi need hydrolase enzymes, such as cellulases and hemicellulases, for breaking down the skeletal lignocellulosic polysaccharides (cellulose and hemicellulose). Second, they need an excellent extracellular ligninolytic system for degradation or modification of lignin [2]. Cu-containing laccases and heme peroxidases (such as LiP, MnP, VP and Dye-decolorizing peroxidase) are the most prevalent fungal ligninases [90]. Fungal accessory enzymes support these main lignin-degrading enzymes [1]. Fungi are the only widely known organisms able to degrade lignin. Three types of fungi degrade the lignin classified by the type of rot they are associated with, as shown below in Figure 6.

### 9.1. White-Rot Fungi

**Figure 7:**
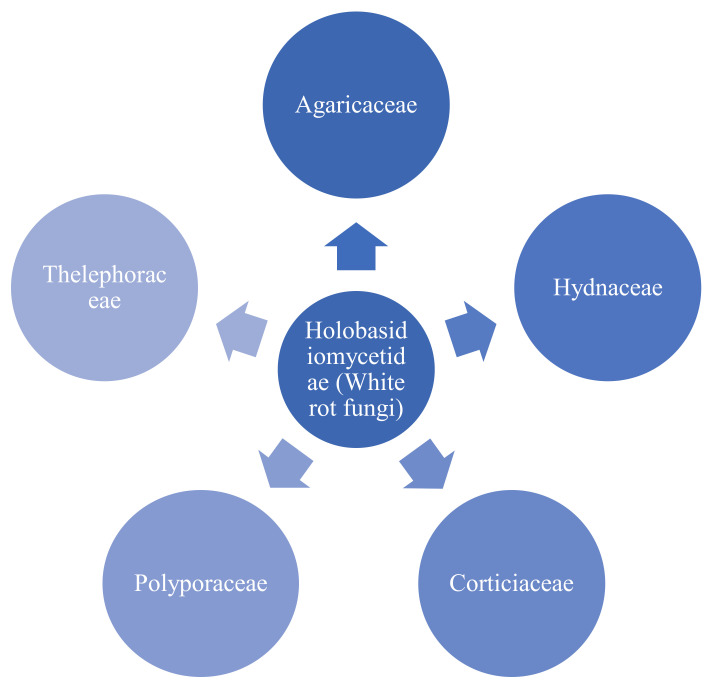
Members of Holobasidiomycetidae (WRF) involved in white-rot-based lignin degradation.

White-rot fungi (WRF) can decompose structural constituents of wood, such as cellulose and lignin, and they are natural producers of extracellular oxidative enzymes [75]. Among the Holobasidiomycetidae are numerous WRF species belonging to various fungal families, shown below in Figure 7 [91].

A few ascomycetes belong to the Xylariaceae family, which causes wood decay similar to that of white-rot. WRF is the most efficient natural lignin degrader among all the ligninolytic microbial groups [91]. WRF fully degrades all structural constituents of lignin, forming carbon dioxide and water as the end products under appropriate environmental conditions. They grow mostly on hardwoods and, due to the presence of syringyl lignin, more efficiently decay hardwood than softwood. In culture, most white-rot fungi show phenoloxidase activity. The phenoloxidase activity along with extracellular peroxidases and laccases creates extracellular colored compounds from phenolic substrates. Ascomycotina and fungi imperfecti are true representatives of eubacteria and actinomycetes, and are well-known lignin-degraders. Ruel et al. observed that there was a modification in the morphology of the lamellar structure of spruce wood lignin. There was also the formation of granulated modified lignin at a distance of up to 2–3 picometers from hyphae of Sporotrichum pulverulentum, which is an anamorphic form of Phanerochaete chrysosporium [92]. At present, among all the ligninolytic systems of WRF, P. chrysosporium-based is the best understood and most widely studied. Recently, Cerro et al., 2021 reported a detailed investigation on the intracellular catabolic pathways for lignin in WRF. The investigators concluded that whether WRF uses lignin as a source of carbon is still controversial. In the present study, investigators used ^13^C isotope labelling, systems biology methods and in vitro enzyme assay to confirm that both fungi (Trametes versicolor and Gelatoporia subvermispora) use carbon from the lignin. After detailed analysis it was found that each utilizes T. versicolor and G. subvermispora 4-HBA and vanillic acid as carbon sources. Such research may contribute to global carbon recycling in soil ecosystems and may further establish a foundation for using WRF in the depolymerization of lignin and its bioconversion into by-products [42].

### 9.2. Brown-Rot Fungi (BRF)

Brown-rot fungi are basidiomycetes evolved from WRF. They are most common in softwood and generally lack phenol oxidase activity [77]. Brown-rot fungi are the wood-rotting fungi that can decay and eliminate wood carbohydrates and leave a remnant of “modified lignin”, which is characteristically brown and has the same mass as the lignin in the wood [78]. Brown-rotters, being mostly basidiomycotina, are taxonomically comparable to white rotters. The brown-colored modified lignin (lignin residue left in brown-rotted wood) is called “enzymatically liberated lignin” [78]. Certain ascomycotina and various fungi imperfecti have the potential to degrade wood under very moist conditions, [93]. These fungi are in many genera, including *Paecilomyes* sp., *Xylaria* sp., *Stachybotrys* sp., *Humicola* sp., *Emericellopsis minima*, *Pestalotia multidea*, *Acremoniella* sp., *Chaetomium globosum*, *Preussia* sp., *Graphium* sp., *Papulospora* sp., *Allescheria* sp., *Cytosporella* sp., *Pestalozzia* sp., *Thielavia* sp. and *Sporocybe* sp. Soft-rot fungi (SRF) can reduce the polysaccharides in wood, but lignin degradation is slow and incomplete [78]. The lignin in brown-rotted wood can be partially degraded by demethylation, partial oxidation and depolymerization, but complete degradation has not been achieved [2].

### 9.3. Soft-Rot Fungi (SRF): Soft Wood

Soft-rot is another form of wood decay that is carried out by ascomycetous fungi [94]. SRF favorably degrade wood carbohydrates, and few of them have established a noteworthy ability to mineralize lignin. In highly moist conditions, some species of ascomycetes and imperfect fungi attack the wood widely. This form of rot exhibits a softening of wood tissue together with a substantial reduction in weight. SRF infiltrate the secondary wall of the wood cell and lead to the formation of cylindrical cavities where the propagation of hyphae takes place. SRF are found amongst numerous genera, such as *Allecheria, Monodictys, Chaetomium, Cephalosporium, Papulospora, Graphium, Paecilomyces and Thielavia*. There are six SRF that degrade lignin in alder, poplar and pine woods, while all the fungi except *Paecilornyces* and *Allescheria* favorably attack the polysaccharides in wood [78,86,87].

## 10. Soil Fungi as Lignin Degraders

Degradation of lignin by fungal cultures (including *P. ostreatus, C. versicolor, E. nidulans, A. wentii, A. terrus, A. niger, C. globosum, Trichoderma viride and T. harzianum*) was tested by oxidative process with phenol oxidase as the key enzyme after a 7-day incubation period [2,16]. The fungi which degraded lignin consisted of ascomycetes (*T. reesei*), basidiomycetes (white-rot and *P. chrysosporium*) and BRF (*Fomitopsis palustris*) [95]. This group comprises mainly the strains of *Aspergillus*, *Fusarium*, *Endoconidiophora* and *Alternaria*. Most of the previous research work conducted on mixed fungal species showed that soil-dwelling fungi can degrade lignin. Work performed by Gulyas (1967) revealed that the pure strains of *Penicillium* and *Fusarium* degraded about 20% of lignin in wheat straw, out of which only 11% could be isolated. Waksman and Hutchings (1936) incubated phenol lignin with a mixed culture containing *Fusarium* and *Alternaria* and reported approximately 25% reduction in lignin, which could be collected by extraction of the incubation medium [95]. An efficiency of about 10–65% was achieved by Fischer (1953) for the degradation of phenol lignin in a liquid medium by using numerous fungi imperfecti [96]. All the fungi that degrade lignin by using various enzymes are summarized below in Table 1.

## 11. Lignin-Degrading Bacteria

Bacterial wood decomposition was primarily measured for forestry rather than biotechnological applications. Genomic and proteomic examination of lignin-reducing bacteria long ago established deficiencies of VP, MnP and LiP enzymes, while bacterial laccases and DyP-type peroxidases have been recognized [89]. DyP-type peroxidases are less composite than the other heme peroxidases and are widespread among bacteria, including the extracellular enzyme systems of *Rhodococcus jostii* and *Thermobifida fusca*. Numerous bacterial laccases as well as several laccase-like, multi-copper oxidases (as in *Sinorhizobium morelense* and *Agromyces salentinus*) are also well-known. Even if knowledge of bacterial peroxidases and laccases has improved recently, additional—as yet undiscovered—enzymes may be necessary for bacterial lignin degradation, for example, oxidases for the making of H_2_O_2_ [1]. The lignin-degrading bacteria secluded from the soil are actinomycetes, α-proteobacteria and γ-proteobacteria. By the mid-1980s, bacterial lignin degradation mechanisms from *Actinomycetes* and *Pseudomonas* species were studied. The bacteria which degrade lignin are comprised of *Actinomycetes* such as *Sphingomonas paucimobilis* SYK-6, *Nocardia, Streptomyces viridosporus* T7A and *Rhodococcus,* which, when grown on lignocellulose, create extracellular peroxidases that degrade both the lignin and carbohydrate components of lignocelluloses. The ligninolytic bacteria were segregated from various natural niches, such as soil, sewage and compost. These isolates were able to decrease the basic dye and were confirmed to be potent lignin degraders. The study of growth rate in different media demonstrated that the isolated bacteria were able to make use of lignin as their only carbon source [114]. The organization of bacteria within rotting wood is well-recognized. Wide-ranging actinomycetes (e.g., *Nocardia, Streptomyces, Thermomonospora* and *Micromonospora*) and eubacteria (e.g., *Pseudomonas, Acinetobacter, Xanthomonas, Bacillus* and *Aeromonas*) degrade a variety of extracted lignin and ‘C-labeled dihydroxyl phenol (DHP). Recently Rashid and Bugg (2021) observed the enhanced biocatalytic biodegradation of lignin by a combination of lignin-degrading enzymes (isolated from bacteria) and accessory enzymes. Here the authors used a set of three bacterial DyP-type peroxidase enzymes from *Ps. fluorescens, Comamonas testosteroni* and *Agrobacterium* sp., two bacterial multi-copper oxidase enzymes CueO from *Ochrobactrum* sp. and CopA from *Ps. putida* and *Sphingobacterium* sp. The concentration of specific products obtained increased in the presence of accessory enzymes, and the overall conversion of lignin into low-molecular-weight products increased by using the combination of Agro DyP/Agro LigE [115]. All the bacteria that degrade lignin by using various enzymes are summarized below in Table 2.

## 12. Mechanism of Lignin Biodegradation

Polymers such as lignin can be biologically degraded by extracellular means during their preliminary stages [131]. During microbial degradation, lignin acidification and ending of the discharge of CO_2_ occurs within the network of thread-like mycelium in fungus [15]. Consequently, the extracellular responses should disintegrate lignin into pieces that can diffuse to the hyphae crossing the cell membranes. In contrast to the additional biopolymers, the lignin monomers are combined via non-hydrolyzed carbon-carbon and ether bonds. The physiological and chemical proofs indicate that degradation of lignin is principally oxidative, though reduction reactions could similarly contribute [124]. The oxidation process of lignin includes unrestricted phenolic groups that would undergo further polymerization [132]. Nevertheless, a few low-molecular-weight pieces are similarly discharged. The lignin-degrading fungi are capable of maintaining polymerization vs. depolymerization stability in favor of disintegration by glycosylation, methylation or by eliminating the lightweight fragments from the reaction combination [2]. In ligninolytic cultures, the DHPs can be rapidly polymerized before depolymerization and mineralization. Lignin has an irregular and complex structure due to the random polymerization process during synthesis. In lignin, the diverse nature of the inter-unit linkages, the occurrence of the asymmetric, stereoisomeric forms at the Cα- and/3-carbons, along with inconsistency within its organization perpetuate challenges for the ligninolytic fungus to create enzymes which enable the break-down process. The fluids developed inside a xylophagous fungus contain enzymes of little selectivity that start the oxidative reactions in lignin but do not direct them. This process was named “the combustion of enzymes” by Kirk and Farrell. They explained that the lignin is triggered by the enzymes to control the energy block and initiate a thermodynamically preferred oxidative disintegration without any control of the reaction pathway [133]. During lignin degradation, water-soluble intermediates can be perceived in either submerged liquid fermentation or solid-state fermentation [134]. Aqueous DHP intermediate deprivation is predominantly oligomeric and has an extensive molecular mass distribution. From decayed wood, partly degraded lignin can be removed using polar organic solvents [135]. Most of this extracted material is oligomeric or polymeric. The biodegradation of lignin does not progress by an arranged exclusion of the peripheral subunits as single ring composites. It includes oxidation of the aromatic rings and side chains inside the polymer, thus increasing the polymer core solubility and hydrophilicity altogether. The unsystematic behavior of the degradation process settles the idea of enzymatic combustion.

## 13. Significance of Lignin Biodegradation

Lignin degradation has garnered immense consideration from various scientists. Enzymes from focused microbes, including fungi and bacteria, that can digest lignin and consume carbon derived from lignin as a food source have been reviewed in the current study [21]. Biodegradation of lignin is accomplished by several microbes that exist in the soil and biomass of plants, and that create ligninolytic enzymes, such as VP, DyP, MnP and LiP [12]. In the coming years, novel lignin-degrading microorganisms and detailed exploration of their biochemistry, proteomics and genomics, will reveal the roles of ligninolytic enzymes [76]. Lignin degradation is exemplified specifically by the capability of white-rot fungi to attain a great extracellular oxidative regimen with little substrate specificity. The biodegradation of lignin as a part of litter decomposition is a significant procedure that has severe impact on human wellbeing. Enhancements in the biodegradation of lignin through optimization of fungal and enzymatic treatments would have widespread impact, affecting sectors such as agriculture, bioremediation and paper and pulp industries [16]. The pathways of naturally occurring degradation of lignin are essential for the expansion of actual bioprocesses centered on enzymatic lignin degradation. Further research work is necessary to recognize the degradation of lignin by bacterial species. The lignin degradation capability of bacterial DyP-type peroxidases and the potential to create them—more-easily than fungal peroxidase—by recombinant expression schemes makes them attractive candidates for the utilization of lignin [136]. The degradation of lignin is followed by sanitization of the reaction mixtures, comprising the elimination of remaining partly tarnished or non-tarnished lignin, which can be used in the reaction to obtain better lignin alteration rates and total procedure effectiveness [1]. Several household and agricultural waste products consist of lignocelluloses-cellulose, hemicellulose and lignin [137]. Cellulose is the major constituent, followed by hemicellulose and lignin. The lignin and hemicellulose cover the cellulose chains, creating a blockade that prevents the entrance of moisture and cellulose-degrading enzymes. Usually a thermo-chemical technique is utilized to treat the lignocellulosic biomass, but it is difficult to process and very expensive. Lignin biodegradation is a vital step for carbon recycling in land ecosystems [4]. The transformation of organic materials into humus by decomposers, such as microorganisms, is known as humification and changes the properties of the soil. Hence lignin biodegradation increases soil fertility [4].

## 14. Modern Applications of Lignin Micro- and Nanoparticles

**Figure 8:**
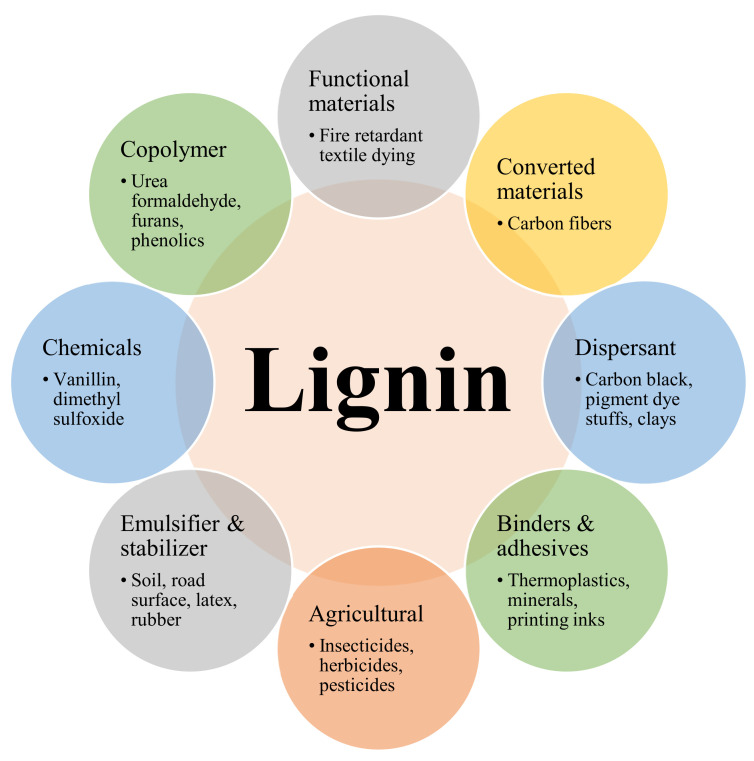
Potential applications of lignin.

Lignin exploration, including its uses, has been going on for many years. Much research has described the prospect of using lignin as a high-value product. Lignin has numerous prospective industrial applications due to its low toxicity and high adaptability [138]. There are several high-value lignin properties, including bulk accessibility, cost-effectiveness and the rising need for bio-based and renewable elements. Lignin can be utilized efficiently for dye or tanning agent dispersal. The dynamic groups present in lignin, such as organosulfur and amino, help effectively scatter dye elements homogenously in aqueous solutions [139]. The utilization of lignin has been restricted to a small number of low-value uses, as shown in Figure 8. The main lignin-derived ingredients, useful products and their applications have been highlighted in Table 3.

The burning of lignin is broadly accomplished in the pulp and paper industries to produce electricity. Lignin is used very efficiently (~90%) as a fuel for the generation of electricity. Lignin combustion in combination with coal is widely applied as fuel for the pulping tank [25]. The lignin salts are particularly water-soluble and are generally utilized as a binder in water-based dye printing [140]. Similarly, lignin-extracted binders may be applied within a silicon anode, which is the best anode substance for lithium-ion batteries [25].

### Lignin-Based Cement, Phenolic Compounds, Carbon Materials and Hydrocarbon Compounds

Lignin-based cement is cost-effective for cement upgradation, as has been described in numerous research investigations [4]. The modified form of lignin produces highly effective concrete, with increased strength, simplified grating and less harm to exterior barriers caused by moisture and acid rain [141]. Innovative applications for lignin products are emerging in diverse research areas. Lignin is identified as a building block for various significant chemicals. Current techniques are mainly dedicated to lignin depolymerization, synthesis of chemically reactive portions, hydroxyl group functionalization and manufacturing of lignin implant copolymers [142]. There are a few innovative applications of lignin that have been described, including lignin-based products, such as carbon fibers, phenolic and oxidized products [143]. Lignin likely has applications in polymeric and polyelectrolytes substances, as it can be significantly extended to produce larger monomers for incorporation into polymers. Current advances seek to optimize lignin reactivity to improve its usage in a diverse range of macro-molecules, such as carbon fiber, polymer alloy, polyurethane and filler [142,144]. Carbon fiber is extensively applied in different areas, such as athletics and the aeronautical business. The automotive industry might also be an obvious area for carbon fiber to substitute steel. Lignin-based carbon substances have been utilized as strengthening in advanced energy-storing and electro-chemical applications, as absorbent ingredients and for refining gas from organic and inorganic contaminants [143]. Lignin may be converted into phenolic composites by synthesizing original chemical sites via hydroxyalkylation. This process includes phenolation, demethylation and methylation. Phenols are reactive acidic-natured compounds because of the occurrence of the −OH group and facilitate the oxidation of phenolic compounds to produce polymers. The biological synthesis of lignin contributes to various forms of phenolic mixtures. Phenols are manufactured by benzene, which is generally petroleum-based. The phenol products can be utilized in the manufacturing of cosmetic [138]. Lignin can be used to make hydrocarbons in a very cost-effective way. Hydrocarbons can be obtained using heterogeneous processes from Klason birch lignin, organosolv, wood sawdust and acidolysis carried out by enzymes [145], and their making is highly dependent on the temperature, catalyst and feed-stock. Ecofriendly thermoset resin polyurethane can be prepared using lignin. It has an exceptional power-to-weight ratio (WR), energy absorption enactment and also has significant usages in the construction of ships, furniture insulation, automobile business and packing trade [146]. The current advancement in nanotechnology has revealed a strong association between the structure of material, properties and performance. Nanoscale lignin alteration to design high-value ingredients is still in the beginning phase. Many research investigations have noted the difficult transition from macromolecular lignin structure to nanoparticles, nanotubes and nanofibers. This methodology supports converting lignin into a more uniform size and shape.

## 15. Lignin-Derived Polymers

Lignin has immense potential as a raw substance for the development of various materials. Extensive usage of polymers and chemicals derived from biomass is requisite for ecological sustainability and protection [149]. Currently, lignin is applied in low-end markets, such as fuel or cement supplements and also the pulp and paper industries [8]. The physicochemical properties and uniqueness of lignin make it an excellent candidate for the development of novel polymeric constituents. Lignin derivatization with the help of polymer chemistry has developed as a very precious path to enhance its thermochemical properties and chemical functionalization to accomplish special usages. The valorization of lignin using polymeric conversions can be categorized into diverse classes, as shown in Figure 7. Lignin is used as monomers to form polymers, and lignin graft polymers are given focus. The well-ordered polymerization approaches allow the control of polymer configurations, functionalities and architectures which enable the improvement of innovative constituents with modified physicochemical properties [7]. These versatile methods are used to produce precise polymers with regulated molecular weight, fine molecular dispersal and site-specific functionality. Some examples are: atom transfer radical polymerization (ATRP) [150], nitroxide-mediated polymerization (NMP) [151], reversible addition fragmentation chain transfer (RAFT) [152], ring-opening polymerization (ROP), ring-opening metathesis polymerization (ROMP) [7] and acyclic diene metathesis (ADMET) [153]. Additionally, versatile methods also permit the production of high grafting densities on graft polymers and control over end-group composition. Numerous reports explore the use of these approaches to prepare lignin-based biopolymers; it must be acknowledged that there are plentiful opportunities for advanced investigation.

Recently Kim and Chung (2021) reported the synthesis and characterization of lignin-graft poly(ethylene-brassylate) which is actually a biobased polyester with remarkable mechanical properties. Here the biosynthesized LiG was surface modified by using chemical sebacic acid in order to add a carboxylic group. The researchers obtained a condensed copolymer of lignin and poly(ethylene brassylate) (PEB), which improved several physical properties of LiG.

Bass and Epps (2021) provided a detailed overview on the latest efforts towards creating performance-enhanced, LiG-based polymers. The authors have emphasized the possibilities and challenges for utilization of LiG blends and composites, thermosets, thermoplastics and vitrimers [154].

## 16. Applications of Lignin in Medicine

There are several reported works where lignin has been used for various biomedical applications either directly or in the form of composite materials. The major advantage of using LiG in this field is their biocompatible and biodegradable nature and easy availability. Lignin is considered antioxidant, antibacterial, antiviral, biodegradable, biocompatible and nontoxic and can thus be used as a medicine against many diseases. Additionally, it has also been used for drug delivery and tissue engineering. LiG NPs are widely applied in the biomedical field, especially for coating of biomedical devices, gene delivery and personal health care [155]. Larraneta et al., 2018, synthesized LiG hydrogels and, after characterization, applied them as drug-eluting antimicrobial coatings for medical devices to prevent the growth of microbial pathogens [27]. Numerous investigators have used LiG and LiG NPs for drug delivery, especially for anticancer drugs. For instance, Garg et al., 2022, described in detail the application of LiG NPs in the delivery of anticancer drugs [156].

## 17. Present Challenges

There is very little information in the literature about the degradation of lignin in the environment. Lignin degradation is a complex process [115], and multidimensional scientific investigation should be undertaken to understand it. Moreover, there is a surprising lack of research on the synthesis of lignin nanoparticles, especially from lignin-rich waste materials. It is well known that fungi, such as brown-rot, soft-rot and white-rot, and a few bacteria are involved in mineralization of lignin, but the exact mechanism is hardly known [157]. For example, brown-rot fungi perform incomplete degradation of lignin, whereas as white- and soft-rot fungi can degrade lignin completely. For some of the microbial lignin degraders the detailed enzymatic pathway is not known; this knowledge would allow the biodegradation pathway to be made more efficient, more effective and faster. This can be made possible by applying certain molecules, such as nanoparticles. Another question relates to whether the fungi are utilizing lignin as their sole source of carbon. If yes, then in which form is the carbon utilized. Cerro et al., 2021, performed ^13^C isotope labelling and provided very detailed intracellular lignin degradation pathways [42]. Apart from these, there are many more current challenges which need to be addressed in more detail in this domain. Some of the lignin biodegraders have a very slow rate of degradation [158]; this is one more challenge for the investigators in this field, which may increase the speed of lignin degraders in the ecosystem and prevent solid-waste-based pollution arising from agricultural and industrial sources.

The current applications of lignin particles in bulk form is not effective either in research or in medicine as drug delivery, so more in-depth research is needed in the synthesis of lignin nanoparticles, especially from lignin-rich waste materials. Further investigation is required in the development of value-added products from agricultural and industrial lignin waste. Applicability of such lignin products could be enhanced by surface functionalization. Gao et al., 2021, showed present use as well as future prospects of lignin-based materials in the biomedical field [155]. 

## 18. Conclusion and Future Perspectives

There is very little information regarding the degradation of lignin in soil. Lignin degradation has received significant consideration from different researchers. Some research is exploring the productive deconstruction and degradation of complex lignin molecules. The major microbial degraders of lignin in our ecosystem are white-, brown- and soft-rot fungi and soil fungi, which produce several extracellular enzymes, such as laccases, lignin peroxidases (LiP), manganese-dependent peroxidase (MnP), dye-decolorizing peroxidase (DyP) and versatile peroxidase for the effective degradation of lignin through the generation of free radicals. The detailed pathways, along with all the enzymes and molecules involved in the degradation of lignin by microorganisms can be made efficient and rapid by introducing nanotechnology and nanoparticles. Lignin nanoparticles offer an adaptable platform for significant applications. The synthesis of LiG NPs by biological routes will be ecofriendly, and the NPs will act as potential candidates for drug delivery in medicine. Much current research has shown that both lignin and lignin nanoparticles have promising applications in the medical field, especially in drug and gene delivery. Though lignin and lignin nanoparticles are biodegradable, their utilization rate is very low in the fields of medicine and research. Surface modifications by various functional groups will increase their application in medicine, especially drug delivery. Formation of magnetic nanoparticles and lignin-hybrid-based nanocomposites could be a boon to medicine and cancer treatment, as the functional groups would allow loading with multiple particles, and magnetic particles could respond to magnetic fields. Due to their biodegradable and biocompatible nature, they could be the most desirable material for the loading of anticancer drugs. Various challenges are encountered by researchers in preparing the chemicals from lignin, which may end up with hazardous chemicals in the environment. Thus, more focused research is required for enzyme-based synthesis of lignin-based chemicals, lignin nanoparticles and other value-added materials. The development of such value-added products from waste rich in lignin will not only minimize the solid waste, but will also provide an alternate and economical material for the synthesis of lignin. In the future, it will be necessary to develop alternate preparation methods for lignin-based nanoparticles, nanocomposites and hybrid materials, which are not only low-cost, sustainable, eco-friendly and easier for large-scale production, but should also be biocompatible and surface-modified, especially for medical applications. Lignin’s antimicrobial properties need to be explored more by extensive research in this filed, which may provide a replacement for the current, toxic antimicrobial products. Additionally, to expand the application performance, special functional groups can be introduced to improve the thermal stability, adsorption capacity, conductivity, magnetic and optical properties of lignin-based nanoparticles.

## Figures and Tables

**Table 1:** Fungi and their lignolytic enzymes in biological degradation of lignin.

Enzyme	Fungi	Reference
DyP	*Auricularia auricular-judae*	[97]
LiP	*P. chrysosporium*	[98]
	*Phlebia radiata*	[99]
	*P. tremellosa*	[100]
MnP	*Phanerochaete sordida*	[101]
	*P. chrysosporium*	[102]
	*Trametes versicolor*	[103]
	*Ceriporiopsis subvermispora*	[104]
LaC	*P. radiata*	[105]
	*C. subvermispora*	[106]
	*Pleurotus eryngii*	[107]
	*T. versicolor*	[108]
	*T. hirsuta*	[109]
	*T. ochracea*	[110]
VP	*P. eryngii*	[111,112]
	*Pleurotus ostreatus*	[112]
	*Bjerkandera fumosa*	[113]

**Table 2:** Bacteria and their lignolytic enzymes in biological degradation of lignin.

Lignolytic Enzyme	Bacteria	Reference
DyP A	*Amycolatopsis* sp.	[12]
	*E. coli*	[116]
	*Rhodococcus jostii*	[117]
	*Streptomyces viridosporus*	[117,118]
	*S. coelicolor*	[119]
	*Thermobifida fusca*	[120]
	*T. fusca YX*	[120]
DyP B	*Escherichia coli*	[121]
	*Pseudomonas* sp.	[122]
	*R. jostii*	[117]
	*S. coelicolor*	[89]
Laccase	*Bacillus atrophaeus*	[123]
	*B. licheniformis*	[124]
	*B. pumilus*	[125]
	*B. subtilis*	[12]
	*S. coelicolor*	[12]
	*S. griseus*	[126]
	*S. ipomoea*	[127]
	*S. lavendulae*	[128]
	*Streptomyces cyaneus*	[129]
	*Thermus thermophilus*	[130]
DyP-type peroxidase	*Ps. fluorescens*, *Comamonas testosteroni* and *Agrobacterium* sp.	[115]

**Table 3:** Categories of lignin-derived products and their applications.

Category	Type	Products	Applications	References
Aromatic macromolecules and fine Chemicals	Klason (Kn), kraft lignin (KL), organosolv (OGs)	Lignin monomers and dimers, aromatic phenols, alkyl phenols, aromatic aldehydes, aromatic alcohols, acids, aryl ketones, antioxidants, dispersants, polyurethanes, phenolic resins, vanillin	Industrial chemicals, bio-based adhesives, multifunctional materials, building blocks for bio-based products	[145]
Polymer and nanomaterials	KL, OGs, straw lignin (SL)	3D printing resin (cationic surfactant), scaffolds, lignin nanotubes, hydrogels, lignin nanotubes	Biomedical applications, tissue engineering, drug delivery	[147]
Carbon materials, biofuels	KL, sulfite, soda, OGs	Biochar, bio-oil, syngas, activated carbon, carbon fibers, carbon black	Light-weight polymer composites, adsorbents, electrochemical devices, automotive	[148]
Specialized applications	KL, sulfite, soda, OGs	Soil conditioner, controlled release agent in fertilizers and pesticides, sequestering agent, contaminant absorbent, fire retardant	agriculture, textiles, soil reclamation, water purification, fire suppression	[145]

## Data Availability

Not applicable.

## Abbreviations

ATRP	atom transfer radical polymerization
ADMET	acyclic diene metathesis
BRF	brown-rot fungi
DHP	di hydroxyl phenol
FTIR	Fourier transform-infrared spectroscopy
LC	lignocellulose
LCBM	lingocellulosic biomass
Lac	laccase
LiP	lignin peroxidases
LNPs	lignin nanoparticles
DyP	dye-decolorizing peroxidase
KL	Kraft lignin
LiG	lignin
MnP	manganese-dependent peroxidase
MLG	monolignols
NMP	nitroxide mediated polymerization
NMR	nuclear magnetic resonance spectroscopy
NPs	nanoparticles
OGs	organosolv
PEB	poly(ethylene brassylate)
RAFT	reversible addition fragmentation chain transfer
ROP	ring-opening polymerization
ROMP	ring-opening metathesis polymerization
SRF	soft-rot fungi
THF	tetrahydrofuran
UV-Vis	ultraviolet visible spectroscopy
VP	versatile peroxidase
WRF	white-rot fungi

## References

[1] Arapova O.V., Chistyakov A.V., Tsodikov M.V., Moiseev I.I. (2020). Lignin as a Renewable Resource of Hydrocarbon Products and Energy Carriers (A Review). Pet. Chem..

[2] Janusz G., Pawlik A., Sulej J., Swiderska-Burek U., Jarosz-Wilkolazka A., Paszczynski A. (2017). Lignin degradation: Microorganisms, enzymes involved, genomes analysis and evolution. FEMS Microbiol. Rev..

[3] Saini J.K., Saini R., Tewari L. (2015). Lignocellulosic agriculture wastes as biomass feedstocks for second-generation bioethanol production: Concepts and recent developments. 3 Biotech.

[4] Ruiz-Dueñas F.J., Martínez Á.T. (2009). Microbial degradation of lignin: How a bulky recalcitrant polymer is efficiently recycled in nature and how we can take advantage of this. Microb. Biotechnol..

[5] Arora A., Nandal P., Singh J., Verma M.L. (2020). Nanobiotechnological advancements in lignocellulosic biomass pretreatment. Mater. Sci. Energy Technol..

[6] Vanholme R., Demedts B., Morreel K., Ralph J., Boerjan W. (2010). Lignin biosynthesis and structure. Plant Physiol..

[7] Ganewatta M.S., Lokupitiya H.N., Tang C. (2019). Lignin Biopolymers in the Age of Controlled Polymerization. Polymers.

[8] Li T., Takkellapati S. (2018). The current and emerging sources of technical lignins and their applications. Biofuel Bioprod. Biorefin..

[9] Rojas-Downing M.M., Nejadhashemi A.P., Harrigan T., Woznicki S.A. (2017). Climate change and livestock: Impacts, adaptation, and mitigation. Clim. Risk Manag..

[10] Taha M., Foda M., Shahsavari E., Aburto-Medina A., Adetutu E., Ball A. (2016). Commercial feasibility of lignocellulose biodegradation: Possibilities and challenges. Curr. Opin. Biotechnol..

[11] Branco R., Serafim L., Xavier A. (2018). Second Generation Bioethanol Production: On the Use of Pulp and Paper Industry Wastes as Feedstock. Fermentation.

[12] Datta R., Kelkar A., Baraniya D., Molaei A., Moulick A., Meena R., Formanek P. (2017). Enzymatic Degradation of Lignin in Soil: A Review. Sustainability.

[13] Bonugli-Santos R., Durrant L., Silva M., Sette L. (2010). Production of laccase, manganese peroxidase and lignin peroxidase by Brazilian marine-derived fungi. Enzym. Microb. Technol..

[14] Su Y., Xian H., Shi S., Zhang C., Manik S.M.N., Mao J., Zhang G., Liao W., Wang Q., Liu H. (2016). Biodegradation of lignin and nicotine with white rot fungi for the delignification and detoxification of tobacco stalk. BMC Biotechnol..

[15] Kumar A., Chandra R. (2020). Ligninolytic enzymes and its mechanisms for degradation of lignocellulosic waste in environment. Heliyon.

[16] Dashtban M., Schraft H., Syed T.A., Qin W. (2010). Fungal biodegradation and enzymatic modification of lignin. Int. J. Biochem. Mol. Biol..

[17] Ahorsu R., Medina F., Constantí M. (2018). Significance and Challenges of Biomass as a Suitable Feedstock for Bioenergy and Biochemical Production: A Review. Energies.

[18] Naseem A., Tabasum S., Zuber M., Ali M., Noreen A. (2016). Lignin-derivatives based polymers, blends and composites: A review. Int. J. Biol. Macromol..

[19] Takkellapati S., Li T., Gonzalez M.A. (2018). An Overview of Biorefinery Derived Platform Chemicals from a Cellulose and Hemicellulose Biorefinery. Clean Technol. Environ. Policy.

[20] Järvinen J., Taskila S., Isomäki R., Ojamo H. (2012). Screening of white-rot fungi manganese peroxidases: A comparison between the specific activities of the enzyme from different native producers. AMB Express.

[21] Brebu M., Vasile C. (2010). Thermal degradation of lignin—A Review. Cellul. Chem. Technol..

[22] Bugg T.D., Ahmad M., Hardiman E.M., Rahmanpour R. (2011). Pathways for degradation of lignin in bacteria and fungi. Nat. Prod. Rep..

[23] Saxer S., Portmann C., Tosatti S., Gademann K., Zürcher S., Textor M. (2010). Surface Assembly of Catechol-Functionalized Poly(l-lysine)-graft-poly(ethylene glycol) Copolymer on Titanium Exploiting Combined Electrostatically Driven Self-Organization and Biomimetic Strong Adhesion. Macromolecules.

[24] Blondiaux E., Bomon J., Smoleń M., Kaval N., Lemière F., Sergeyev S., Diels L., Sels B., Maes B.U.W. (2019). Bio-based Aromatic Amines from Lignin-Derived Monomers. ACS Sustain. Chem. Eng..

[25] Bajwa D., Pourhashem G., Ullah A.H., Bajwa S. (2019). A concise review of current lignin production, applications, products and their environment impact. Ind. Crops Prod..

[26] Bonilla A.F., Bonilla D.A. (2021). Synthesis and Characterization of a Novel Lignin-Based Biopolymer from Ulex europaeus: A Preliminary Study. J.

[27] Larrañeta E., Imízcoz M., Toh J.X., Irwin N.J., Ripolin A., Perminova A., Domínguez-Robles J., Rodríguez A., Donnelly R.F. (2018). Synthesis and Characterization of Lignin Hydrogels for Potential Applications as Drug Eluting Antimicrobial Coatings for Medical Materials. ACS Sustain. Chem. Eng..

[28] An L., Yu Y.H., Chen J., Bae J.H., Youn D.H., Jeong H.M., Kim Y.S. (2021). Synthesis and characterization of tailor-made zwitterionic lignin for resistance to protein adsorption. Ind. Crops Prod..

[29] Spiridon I., Dascalu I.-A., Coroaba A., Apostol I., Palamaru M.N., Iordan A.R., Borhan A.I. (2021). Synthesis and Characterization of New Ferrite-Lignin Hybrids. Polymers.

[30] Kumar R., Butreddy A., Kommineni N., Reddy P.G., Bunekar N., Sarkar C., Dutt S., Mishra V.K., Aadil K.R., Mishra Y.K. (2021). Lignin: Drug/Gene Delivery and Tissue Engineering Applications. Int. J. Nanomed..

[31] Khan M., Khan A.U., Hasan M.A., Yadav K.K., Pinto M.M.C., Malik N., Yadav V.K., Khan A.H., Islam S., Sharma G.K. (2021). Agro-Nanotechnology as an Emerging Field: A Novel Sustainable Approach for Improving Plant Growth by Reducing Biotic Stress. Appl. Sci..

[32] Yadav V.K., Choudhary N., Ali D., Gnanamoorthy G., Inwati G.K., Almarzoug M.H.A., Kumar G., Khan S.H., Solanki M.B. (2021). Experimental and Computational Approaches for the Structural Study of Novel Ca-Rich Zeolites from Incense Stick Ash and Their Application for Wastewater Treatment. Adsorpt. Sci. Technol..

[33] Yadav V.K., Khan S.H., Choudhary N., Tirth V., Kumar P., Ravi R.K., Modi S., Khayal A., Shah M.P., Sharma P. (2021). Nanobioremediation: A sustainable approach towards the degradation of sodium dodecyl sulfate in the environment and simulated conditions. J. Basic Microbiol..

[34] Yadav V.K., Yadav K.K., Alam J., Cabral-Pinto M.M.S., Gnanamoorthy G., Alhoshan M., Kamyab H., Hamid A.A., Ahmed F.A., Shukla A.K. (2021). Transformation of hazardous sacred incense sticks ash waste into less toxic product by sequential approach prior to their disposal into the water bodies. Environ. Sci. Pollut. Res..

[35] Gnanamoorthy G., Ramar K., Padmanaban A., Yadav V.K., Suresh Babu K., Karthikeyan V., Narayanan V. (2020). Implementation of ZnSnO_3_ nanosheets and their RE (Er, Eu, and Pr) materials: Enhanced photocatalytic activity. Adv. Powder Technol..

[36] Modi S., Prajapati R., Inwati G.K., Deepa N., Tirth V., Yadav V.K., Yadav K.K., Islam S., Gupta P., Kim D.-H. (2022). Recent Trends in Fascinating Applications of Nanotechnology in Allied Health Sciences. Crystals.

[37] Alam J., Yadav V.K., Yadav K.K., Cabral-Pinto M.M., Tavker N., Choudhary N., Shukla A.K., Ali F.A., Alhoshan M., Hamid A.A. (2021). Recent Advances in Methods for the Recovery of Carbon Nanominerals and Polyaromatic Hydrocarbons from Coal Fly Ash and Their Emerging Applications. Crystals.

[38] Yadav V.K., Fulekar M.H. (2020). Advances in Methods for Recovery of Ferrous, Alumina, and Silica Nanoparticles from Fly Ash Waste. Ceramics.

[39] Kumar Yadav V., Suriyaprabha R., Heena Khan S., Singh B., Gnanamoorthy G., Choudhary N., Kumar Yadav A., Kalasariya H. (2020). A novel and efficient method for the synthesis of amorphous nanosilica from fly ash tiles. Mater. Today Proc..

[40] Gupta N., Yadav V.K., Yadav K.K., Alwetaishi M., Gnanamoorthy G., Singh B., Jeon B.-H., Cabral-Pinto M.M.S., Choudhary N., Ali D. (2022). Recovery of iron nanominerals from sacred incense sticks ash waste collected from temples by wet and dry magnetic separation method. Environ. Technol. Innov..

[41] Meng X., Poonia M., Yoo C.G., Ragauskas A.J. (2021). Recent Advances in Synthesis and Application of Lignin Nanoparticles. Lignin Utilization Strategies: From Processing to Applications.

[42] Del Cerro C., Erickson E., Dong T., Wong A.R., Eder E.K., Purvine S.O., Mitchell H.D., Weitz K.K., Markillie L.M., Burnet M.C. (2021). Intracellular pathways for lignin catabolism in white-rot fungi. Proc. Natl. Acad. Sci. USA.

[43] Lu Y., Lu Y.-C., Hu H.-Q., Xie F.-J., Wei X.-Y., Fan X. (2017). Structural Characterization of Lignin and Its Degradation Products with Spectroscopic Methods. J. Spectrosc..

[44] Alzagameem A., Khaldi-Hansen B.E., Büchner D., Larkins M., Kamm B., Witzleben S., Schulze M. (2018). Lignocellulosic Biomass as Source for Lignin-Based Environmentally Benign Antioxidants. Molecules.

[45] Stark N., Yelle D., Agarwal U., Omar F., Sain M. (2015). Techniques for Characterizing Lignin. Lignin in Polymer Composites.

[46] Sette M., Wechselberger R., Crestini C. (2011). Elucidation of Lignin Structure by Quantitative 2D NMR. Chem. Eur. J..

[47] Wang Y., Chantreau M., Sibout R., Hawkins S. (2013). Plant cell wall lignification and monolignol metabolism. Front. Plant Sci..

[48] Achyuthan K.E., Achyuthan A.M., Adams P.D., Dirk S.M., Harper J.C., Simmons B.A., Singh A.K. (2010). Supramolecular self-assembled chaos: Polyphenolic lignin’s barrier to cost-effective lignocellulosic biofuels. Molecules.

[49] Iravani S., Varma R.S. (2020). Greener synthesis of lignin nanoparticles and their applications. Green Chem..

[50] Beisl S., Friedl A., Miltner A. (2017). Lignin from Micro- to Nanosize: Applications. Int. J. Mol. Sci..

[51] Kharissova O.V., Kharisov B.I., Oliva González C.M., Méndez Y.P., López I. (2019). Greener synthesis of chemical compounds and materials. R. Soc. Open Sci..

[52] Myint A.A., Lee H.W., Seo B., Son W.-S., Yoon J., Yoon T.J., Park H.J., Yu J., Yoon J., Lee Y.-W. (2016). One pot synthesis of environmentally friendly lignin nanoparticles with compressed liquid carbon dioxide as an antisolvent. Green Chem..

[53] Gupta A.K., Mohanty S., Nayak S.K. (2014). Synthesis, Characterization and Application of Lignin Nanoparticles (LNPs). Mater. Focus.

[54] Gilca I., Popa V., Crestini C. (2014). Obtaining lignin nanoparticles by sonication. Ultrason. Sonochem..

[55] Malik M.A., Wani M.Y., Hashim M.A. (2012). Microemulsion method: A novel route to synthesize organic and inorganic nanomaterials: 1st Nano Update. Arab. J. Chem..

[56] Matsakas L., Gerber M., Yu L., Rova U., Christakopoulos P. (2020). Preparation of low carbon impact lignin nanoparticles with controllable size by using different strategies for particles recovery. Ind. Crops Prod..

[57] Mattinen M.L., Valle-Delgado J.J., Leskinen T., Anttila T., Riviere G., Sipponen M., Paananen A., Lintinen K., Kostiainen M., Osterberg M. (2018). Enzymatically and chemically oxidized lignin nanoparticles for biomaterial applications. Enzym. Microb. Technol..

[58] Pan M., Xie X., Liu K., Yang J., Hong L., Wang S. (2020). Fluorescent Carbon Quantum Dots-Synthesis, Functionalization and Sensing Application in FoodAnalysis. Nanomaterials.

[59] Mishra P.K., Ekielski A. (2019). The Self-Assembly of Lignin and Its Application in Nanoparticle Synthesis: A Short Review. Nanomaterials.

[60] Schneider W.D.H., Dillon A.J.P., Camassola M. (2021). Lignin nanoparticles enter the scene: A promising versatile green tool for multiple applications. Biotechnol. Adv..

[61] Chen F., Hu X., Tu X., Chen L., Liu X., Tan L., Mao Y., Shi J., Teng X., He S. (2020). High-Yield Production of Lignin-Derived Functional Carbon Nanosheet for Dye Adsorption. Polymers.

[62] Henn A., Mattinen M.-L. (2019). Chemo-enzymatically prepared lignin nanoparticles for value-added applications. World J. Microbiol. Biotechnol..

[63] Nair S.S., Sharma S., Pu Y., Sun Q., Pan S., Zhu J.Y., Deng Y., Ragauskas A.J. (2014). High Shear Homogenization of Lignin to Nanolignin and Thermal Stability of Nanolignin-Polyvinyl Alcohol Blends. ChemSusChem.

[64] Frangville C., Rutkevičius M., Richter A., Velev O., Stoyanov S., Paunov V. (2012). Fabrication of Environmentally Biodegradable Lignin Nanoparticles. Chemphyschem.

[65] Cailotto S., Gigli M., Bonini M., Rigoni F., Crestini C. (2020). Sustainable Strategies in the Synthesis of Lignin Nanoparticles for the Release of Active Compounds: A Comparison. ChemSusChem.

[66] Zhang Z., Terrasson V., Guenin E. (2021). Lignin Nanoparticles and Their Nanocomposites. Nanomaterials.

[67] Agustin M.B., Penttilä P.A., Lahtinen M., Mikkonen K.S. (2019). Rapid and Direct Preparation of Lignin Nanoparticles from Alkaline Pulping Liquor by Mild Ultrasonication. ACS Sustain. Chem. Eng..

[68] Tran M.H., Phan D.-P., Lee E.Y. (2021). Review on lignin modifications toward natural UV protection ingredient for lignin-based sunscreens. Green Chem..

[69] Salentinig S., Schubert M. (2017). Softwood Lignin Self-Assembly for Nanomaterial Design. Biomacromolecules.

[70] Yearla S.R., Padmasree K. (2016). Preparation and characterisation of lignin nanoparticles: Evaluation of their potential as antioxidants and UV protectants. J. Exp. Nanosci..

[71] Rangan A., Manchiganti M.V., Thilaividankan R.M., Kestur S.G., Menon R. (2017). Novel method for the preparation of lignin-rich nanoparticles from lignocellulosic fibers. Ind. Crops Prod..

[72] Juikar S.J., Nadanathangam V. (2020). Microbial Production of Nanolignin from Cotton Stalks and Its Application onto Cotton and Linen Fabrics for Multifunctional Properties. Waste Biomass Valoriz..

[73] Tao J., Chow S.F., Zheng Y. (2019). Application of flash nanoprecipitation to fabricate poorly water-soluble drug nanoparticles. Acta Pharm. Sin. B.

[74] Conner C.G., Veleva A.N., Paunov V.N., Stoyanov S.D., Velev O.D. (2020). Scalable Formation of Concentrated Monodisperse Lignin Nanoparticles by Recirculation-Enhanced Flash Nanoprecipitation. Part. Part. Syst. Charact..

[75] Leonowicz A., Matuszewska A., Luterek J., Ziegenhagen D., Wojtaś-Wasilewska M., Cho N.-S., Hofrichter M., Rogalski J. (1999). Biodegradation of Lignin by White Rot Fungi. Fungal Genet. Biol..

[76] Kärkönen A., Koutaniemi S. (2010). Lignin Biosynthesis Studies in Plant Tissue Cultures. J. Integr. Plant Biol..

[77] Goodell B., Jellison J., Schultz T.P., Militz H., Freeman M.H., Goodell B., Nicholas D.D. (2008). Fungal Decay of Wood: Soft Rot. Development of Commercial Wood Preservatives: Efficacy, Environmental, and Health Issues.

[78] Goodell B. (2003). Brown-Rot Fungal Degradation of Wood: Our Evolving View. ACS Symp. Ser..

[79] Purnomo A., Mori T., Kondo R. (2010). Involvement of Fenton reaction in DDT degradation by brown-rot fungi. Int. Biodeterior. Biodegrad..

[80] Filley T., Cody G.D., Goodell B., Jellison J., Noser C., Ostrofsky A. (2002). Lignin demethylation and polysaccharide decomposition in spruce sapwood degraded by brown rot fungi. Org. Geochem..

[81] Yang J.M., Goring D. (2011). The phenolic hydroxyl content of lignin in spruce wood. Can. J. Chem..

[82] Ohashi Y., Uno Y., Amirta R., Watanabe T., Honda Y., Watanabe T. (2011). Alkoxyl- and carbon-centered radicals as primary agents for degrading non-phenolic lignin-substructure model compounds. Org. Biomol. Chem..

[83] Bjørsvik H.-R., Occhipinti G., Gambarotti C., Cerasino L., Jensen V. (2005). Synthesis of Methoxy-Substituted Phenols by Peracid Oxidation of the Aromatic Ring. J. Org. Chem..

[84] Rosini E., Allegretti C., Melis R., Cerioli L., Conti G., Pollegioni L., D’Arrigo P. (2016). Cascade enzymatic cleavage of the β-O-4 linkage in a lignin model compound. Catal. Sci. Technol..

[85] Zhu Y., Ouyang X., Zhao Y., Jiang L., Guo H., Xueqing Q. (2017). Oxidative depolymerization of lignin improved by enzymolysis pretreatment with laccase. J. Energy Chem..

[86] Chowdhary P., Shukla G., Raj G., Ferreira L.F., Bharagava R. (2019). Microbial manganese peroxidase: A ligninolytic enzyme and its ample opportunities in research. SN Appl. Sci..

[87] Linares N., Magaña-Ortíz D., Guzmán-Ortiz D., Fernández F., Loske A., Gómez Lim M. (2014). Erratum to: High-yield production of manganese peroxidase, lignin peroxidase, and versatile peroxidase in Phanerochaete chrysosporium. Appl. Microbiol. Biotechnol..

[88] Falade A.O., Nwodo U.U., Iweriebor B.C., Green E., Mabinya L.V., Okoh A.I. (2017). Lignin peroxidase functionalities and prospective applications. Microbiologyopen.

[89] De Gonzalo G., Colpa D.I., Habib M.H.M., Fraaije M.W. (2016). Bacterial enzymes involved in lignin degradation. J. Biotechnol..

[90] Sugano Y. (2009). DyP-type peroxidases comprise a novel heme peroxidase family. Cell. Mol. Life Sci. CMLS.

[91] Park J.-H., Pavlov I.N., Kim M.-J., Park M.S., Oh S.-Y., Park K.H., Fong J.J., Lim Y.W. (2020). Investigating Wood Decaying Fungi Diversity in Central Siberia, Russia Using ITS Sequence Analysis and Interaction with Host Trees. Sustainability.

[92] Daniel G. (2014). Fungal and Bacterial Biodegradation: White Rots, Brown Rots, Soft Rots, and Bacteria. Deterioration and Protection of Sustainable Biomaterials.

[93] Savory J. (2008). Breakdown of timber by Ascomycetes and Fungi Imperfecti. Ann. Appl. Biol..

[94] Lee Y.S. (2018). Observation of Soft-Rot Wood Degradation Caused by Higher Ascomyceteous fungi. Mycobiology.

[95] Martinez A.T., Speranza M., Ruiz-Dueñas F., Ferreira Neila P., Camarero S., Guillen F. (2005). Biodegradation of lignocellulosics: Microbial, chemical, and enzymatic as pects of the fungal attack of lignin. Int. Microbiol..

[96] Guiraud P., Steiman R., Seigle-Murandi F., Benoit-Guyod J.L. (1995). Comparison of the toxicity of various lignin-related phenolic compounds toward selected fungi perfecti and fungi imperfecti. Ecotoxicol. Environ. Saf..

[97] Fan X., Zhou Y., Xiao Y., Xu Z., Bian Y. (2014). Cloning, expression and phylogenetic analysis of a divergent laccase multigene family in Auricularia auricula-judae. Microbiol. Res..

[98] Schoemaker H.E., Leisola M.S.A. (1990). Degradation of lignin by Phanerochaete chrysosporium. J. Biotechnol..

[99] Kantelinen A., Waldner R., Niku-Paavola M.-L., Leisola M.S.A. (1988). Comparison of two lignin-degrading fungi:Phlebia radiata andPhanerochaete chrysosporium. Appl. Microbiol. Biotechnol..

[100] Fackler K., Gradinger C., Hinterstoisser B., Messner K., Schwanninger M. (2006). Lignin degradation by white rot fungi on spruce wood shavings during short-time solid-state fermentations monitored by near infrared spectroscopy. Enzym. Microb. Technol..

[101] Hatakka A.I., Uusi-Rauva A.K. (1983). Degradation of 14C-labelled poplar wood lignin by selected white-rot fungi. Eur. J. Appl. Microbiol. Biotechnol..

[102] Bonnarme P., Jeffries T.W. (1990). Mn(II) Regulation of Lignin Peroxidases and Manganese-Dependent Peroxidases from Lignin-Degrading White Rot Fungi. Appl. Environ. Microbiol..

[103] Paice M.G., Reid I.D., Bourbonnais R., Archibald F.S., Jurasek L. (1993). Manganese Peroxidase, Produced by Trametes versicolor during Pulp Bleaching, Demethylates and Delignifies Kraft Pulp. Appl. Environ. Microbiol..

[104] Jensen K.A., Bao W., Kawai S., Srebotnik E., Hammel K.E. (1996). Manganese-Dependent Cleavage of Nonphenolic Lignin Structures by Ceriporiopsis subvermispora in the Absence of Lignin Peroxidase. Appl. Environ. Microbiol..

[105] Kantelinen A., Hatakka A., Viikari L. (1989). Production of lignin peroxidase and laccase by Phlebia radiata. Appl. Microbiol. Biotechnol..

[106] Fernández-Fueyo E., Ruiz-Dueñas F.J., Miki Y., Martínez M.J., Hammel K.E., Martínez A.T. (2012). Lignin-degrading peroxidases from genome of selective ligninolytic fungus Ceriporiopsis subvermispora. J. Biol. Chem..

[107] Camarero S., Martínez M.J., Martínez A.T., Roussos S., Lonsane B.K., Raimbault M., Viniegra-Gonzalez G. (1997). Lignin-degrading enzymes produced by Pleurotus species during solid state fermentation of wheat straw. Advances in Solid State Fermentation.

[108] Knežević A., Milovanovic I., Stajic M., Vukojevic J. (2013). Potential of Trametes species to degrade lignin. Int. Biodeterior. Biodegrad..

[109] Vasina D.V., Pavlov A.R., Koroleva O.V. (2016). Extracellular proteins of Trametes hirsuta st. 072 induced by copper ions and a lignocellulose substrate. BMC Microbiol..

[110] Christopher L.P., Yao B., Ji Y. (2014). Lignin Biodegradation with Laccase-Mediator Systems. Front. Energy Res..

[111] Palma C., Lloret L., Sepúlveda L., Contreras E. (2016). Production of versatile peroxidase from Pleurotus eryngii by solid-state fermentation using agricultural residues and evaluation of its catalytic properties. Prep. Biochem. Biotechnol..

[112] Fernández-Fueyo E., Ruiz-Dueñas F.J., Martínez M.J., Romero A., Hammel K.E., Medrano F.J., Martínez A.T. (2014). Ligninolytic peroxidase genes in the oyster mushroom genome: Heterologous expression, molecular structure, catalytic and stability properties, and lignin-degrading ability. Biotechnol. Biofuels.

[113] Grąz M., Jarosz-Wilkołazka A. (2011). Oxalic acid, versatile peroxidase secretion and chelating ability of Bjerkandera fumosa in rich and limited culture conditions. World J. Microbiol. Biotechnol..

[114] Ruiz-Dueñas F., Lundell T., Floudas D., Nagy L.G., Barrasa J., Hibbett D., Martinez A.T. (2013). Lignin-degrading peroxidases in Polyporales: An evolutionary survey based on 10 sequenced genomes. Mycologia.

[115] Rashid G.M.M., Bugg T.D.H. (2021). Enhanced biocatalytic degradation of lignin using combinations of lignin-degrading enzymes and accessory enzymes. Catal. Sci. Technol..

[116] Fisher A.B., Fong S.S. (2014). Lignin biodegradation and industrial implications. AIMS Bioeng..

[117] Ahmad M., Roberts J.N., Hardiman E.M., Singh R., Eltis L.D., Bugg T.D.H. (2011). Identification of DypB from Rhodococcus jostii RHA1 as a Lignin Peroxidase. Biochemistry.

[118] Lee S., Kang M., Bae J.-H., Sohn J.-H., Sung B.H. (2019). Bacterial Valorization of Lignin: Strains, Enzymes, Conversion Pathways, Biosensors, and Perspectives. Front. Bioeng. Biotechnol..

[119] Majumdar S., Lukk T., Solbiati J.O., Bauer S., Nair S.K., Cronan J.E., Gerlt J.A. (2014). Roles of Small Laccases from Streptomyces in Lignin Degradation. Biochemistry.

[120] Rahman Pour R., Rea D., Jamshidi S., Fülöp V., Bugg T. (2016). Structure of Thermobifida fusca DyP-type Peroxidase and Activity towards Kraft lignin and Lignin Model Compounds. Arch. Biochem. Biophys..

[121] Lambertz C., Ece S., Fischer R., Commandeur U. (2016). Progress and obstacles in the production and application of recombinant lignin-degrading peroxidases. Bioengineered.

[122] Yang C., Yue F., Cui Y., Xu Y., Shan Y., Liu B., Zhou Y., Lü X. (2018). Biodegradation of lignin by *Pseudomonas* sp. Q18 and the characterization of a novel bacterial DyP-type peroxidase. J. Ind. Microbiol. Biotechnol..

[123] Guo H., Lin C., Wang S., Jiang D., Zheng B., Liu Y., Qin W. (2017). Characterization of a Novel Laccase-producing *Bacillus* sp. A4 and its Application in Miscanthus Degradation. BioResources.

[124] Janusz G., Pawlik A., Swiderska-Burek U., Polak J., Sulej J., Jarosz-Wilkolazka A., Paszczynski A. (2020). Laccase Properties, Physiological Functions, and Evolution. Int. J. Mol. Sci..

[125] Reiss R., Ihssen J., Thöny-Meyer L. (2011). Bacillus pumilus laccase: A heat stable enzyme with a wide substrate spectrum. BMC Biotechnol.

[126] Buraimoh O.M., Amund O.O., Ilori M.O. (2016). Kraft lignin degradation by autochtonous streptomyces strains isolated from a tropical lagoon ecosystem. J. Microbiol. Biotechnol. Food Sci..

[127] Ece S., Lambertz C., Fischer R., Commandeur U. (2017). Heterologous expression of a Streptomyces cyaneus laccase for biomass modification applications. AMB Express.

[128] Jing D. (2010). Improving the simultaneous production of laccase and lignin peroxidase from Streptomyces lavendulae by medium optimization. Bioresour. Technol..

[129] Blánquez A., Ball A.S., González-Pérez J.A., Jiménez-Morillo N.T., González-Vila F., Arias M.E., Hernández M. (2017). Laccase SilA from Streptomyces ipomoeae CECT 3341, a key enzyme for the degradation of lignin from agricultural residues?. PLoS ONE.

[130] Miyazaki K. (2005). A hyperthermophilic laccase from Thermus thermophilus HB27. Extremophiles.

[131] Li X., Zheng Y. (2020). Biotransformation of lignin: Mechanisms, applications and future work. Biotechnol. Prog..

[132] Khan A., Nair V., Colmenares J.C., Gläser R. (2018). Lignin-Based Composite Materials for Photocatalysis and Photovoltaics. Top. Curr. Chem..

[133] Kirk T.K., Farrell R.L. (1987). Enzymatic “Combustion”: The Microbial Degradation of Lignin. Annu. Rev. Microbiol..

[134] Kuhad R.C., Kuhar S., Sharma K.K., Shrivastava B., Kuhad R.C., Singh A. (2013). Microorganisms and Enzymes Involved in Lignin Degradation Vis-à-vis Production of Nutritionally Rich Animal Feed: An Overview. Biotechnology for Environmental Management and Resource Recovery.

[135] Hasanov I., Raud M., Kikas T. (2020). The Role of Ionic Liquids in the Lignin Separation from Lignocellulosic Biomass. Energies.

[136] Pollegioni L., Tonin F., Rosini E. (2015). Lignin-degrading enzymes. FEBS J..

[137] Kucharska K., Rybarczyk P., Holowacz I., Lukajtis R., Glinka M., Kaminski M. (2018). Pretreatment of Lignocellulosic Materials as Substrates for Fermentation Processes. Molecules.

[138] Fytianos G., Rahdar A., Kyzas G.Z. (2020). Nanomaterials in Cosmetics: Recent Updates. Nanomaterials.

[139] Fadhel A.Z., Pollet P., Liotta C.L., Eckert C.A. (2010). Combining the Benefits of Homogeneous and Heterogeneous Catalysis with Tunable Solvents and Nearcritical Water. Molecules.

[140] Luo C., Du L., Wu W., Xu H., Zhang G., Li S., Wang C., Lu Z., Deng Y. (2018). Novel Lignin-Derived Water-Soluble Binder for Micro Silicon Anode in Lithium-Ion Batteries. ACS Sustain. Chem. Eng..

[141] Meister J. (2007). Modification of lignin. J. Macromol. Sci.—Polym. Rev..

[142] Wang Y.Y., Meng X., Pu Y., Ragauskas A.J. (2020). Recent Advances in the Application of Functionalized Lignin in Value-Added Polymeric Materials. Polymers.

[143] Gillet S., Aguedo M., Petitjean L., Rita C., Morais A., Lopes A., Lukasik R., Anastas P. (2017). Lignin Transformations for High Value Applications: Towards Targeted Modifications Using Green Chemistry. Green Chem..

[144] Kamara J.M., Heidrich O., Tafaro V.E., Maltese S., Dejaco M.C., Re Cecconi F. (2020). Change Factors and the Adaptability of Buildings. Sustainability.

[145] Cao L., Yu I.K.M., Liu Y., Ruan X., Tsang D.C.W., Hunt A.J., Ok Y.S., Song H., Zhang S. (2018). Lignin valorization for the production of renewable chemicals: State-of-the-art review and future prospects. Bioresour Technol..

[146] Akampumuza O., Wambua P., Ahmed A., Wei l., Qin X.-H. (2015). A review of the applications of bio composites in the automotive industry. Polym. Compos..

[147] Aro T., Fatehi P. (2017). Production and Application of Lignosulfonates and Sulfonated Lignin. ChemSusChem.

[148] Puziy A., Poddubnaya O., Sevastyanova O. (2018). Carbon Materials from Technical Lignins: Recent Advances. Top. Curr. Chem..

[149] Bušić A., Marđetko N., Kundas S., Morzak G., Belskaya H., Ivančić Šantek M., Komes D., Novak S., Šantek B. (2018). Bioethanol Production from Renewable Raw Materials and Its Separation and Purification: A Review. Food Technol. Biotechnol..

[150] Matyjaszewski K. (2012). Atom Transfer Radical Polymerization (ATRP): Current Status and Future Perspectives. Macromolecules.

[151] Tomoeda S., Kitayama Y., Wakamatsu J., Minami H., Zetterlund P.B., Okubo M. (2011). Nitroxide-Mediated Radical Polymerization in Microemulsion (Microemulsion NMP) of n-Butyl Acrylate. Macromolecules.

[152] Gupta C., Washburn N.R. (2014). Polymer-Grafted Lignin Surfactants Prepared via Reversible Addition–Fragmentation Chain-Transfer Polymerization. Langmuir.

[153] Nomura K., Chaijaroen P., Abdellatif M.M. (2020). Synthesis of Biobased Long-Chain Polyesters by Acyclic Diene Metathesis Polymerization and Tandem Hydrogenation and Depolymerization with Ethylene. ACS Omega.

[154] Bass G.F., Epps T.H. (2021). Recent developments towards performance-enhancing lignin-based polymers. Polym. Chem..

[155] Gao W., Kong F., Chen J., Fatehi P., Santos H., Figueiredo P. (2021). Chapter 13—Present and future prospective of lignin-based materials in biomedical fields. Lignin-Based Materials for Biomedical Applications.

[156] Garg J., Nee Chiu M., Krishnan S., Kumar Tripathi L., Pandit S., Farasati Far B., Kumar Jha N., Kumar Kesari K., Tripathi V., Pandey S. (2022). Applications of lignin nanoparticles for cancer drug delivery: An update. Mater. Lett..

[157] Hermosilla E., Rubilar O., Schalchli H., da Silva A.S.A., Ferreira-Leitao V., Diez M.C. (2018). Sequential white-rot and brown-rot fungal pretreatment of wheat straw as a promising alternative for complementary mild treatments. Waste Manag..

[158] Abe M.M., Branciforti M.C., Brienzo M. (2021). Biodegradation of Hemicellulose-Cellulose-Starch-Based Bioplastics and Microbial Polyesters. Recycling.

